# Detection of *A. phagocytophilum* and *E. chaffeensis* in Patient and Mouse Blood and Ticks by a Duplex Real-Time PCR Assay

**DOI:** 10.1371/journal.pone.0074796

**Published:** 2013-09-04

**Authors:** Tuo Dong, Zhangyi Qu, Lijuan Zhang

**Affiliations:** 1 School of Public Health, Harbin Medical University, Harbin, Heilongjiang Province, China; 2 Department of Anaplasma, National Institute for Communicable Disease Control and Prevention, Chinese Centre for Disease Control and Prevention, Beijing, China; The Johns Hopkins University School of Medicine, United States of America

## Abstract

Human granulocytic anaplasmosis (HGA) and human monocytic ehrlichiosis (HME) are emerging, tick-borne, zoonotic infectious diseases caused by *Anaplasma phagocytophilum* and *Ehrlichia chaffeensis*, respectively. Early diagnosis is essential for rapid clinical treatment to avoid misdiagnosis and severe patient outcomes. Simple, sensitive and reliable diagnostic methods are urgently needed. In this study, we developed a duplex real-time PCR assay targeting the *A. phagocytophilum ankA* gene and the *E. chaffeensis* TRP120 gene, respectively. The lowest limit of detection of the duplex real-time PCR assay was 100 copies of the targeted *A. phagocytophilum ankA* gene and the *E. chaffeensis* TRP120 gene per reaction, and the specificity was 100%. Detection in blood DNA samples from the acute stage of illness for 22 HGA cases and 8 HME cases indicated that the duplex real-time PCR assay was more sensitive than the nested PCR assay. The infection of *Citellus* *undulatus* *Pallas* with *A. phagocytophilum* and *E. chaffeensis* was first confirmed in Xinjiang Province and the positive rate was 3.1% for *A. phagocytophilum*, 6.3% for *E. chaffeensis* and 3.1% for co-infection with both pathogens. The rates of *A. phagocytophilum* and *E. chaffeensis* infection of *D* *. silvarum* ticks collected from Shanxi Province were 8.2% and 14.8%, respectively, and the co-infection rate was 3.3%. The rates of *A. phagocytophilum* and *E. chaffeensis* infection in *H. longicornis* ticks collected from Shandong Province were 1.6% and 6.3%, respectively, and the co-infection rate was 1.6%.

## Introduction

Human granulocytic anaplasmosis (HGA) and human monocytic ehrlichiosis (HME) are emerging, tick-borne, infectious diseases caused by the obligate intracellular bacteria *A. phagocytophilum* and *E. chaffeensis*, respectively [1–5]. *A. phagocytophilum* and *E. chaffeensis* have a worldwide distribution, and the prevalence of these infectious diseases has steadily increased in recent years. In the United States, the incidence of *E. chaffeensis* increased from 0.80 to 3.0 cases/10^5^ population per year [6], with a hospitalization rate of 49.0% and a case-fatality rate of 1.9%. Similarly, the seroprevalence of human granulocytic anaplasmosis is as high as 15-36% within the epidemic regions of the United States [7]. Serological and molecular evidence also suggests that human infections by these two pathogens are common in Asian countries, including Korea, Japan and China [8–13].

In China, rickettsial diseases, specifically tick-borne rickettsiosis (e.g., spotted fever, anaplasmosis and ehrlichiosis), are typically under recognized or misdiagnosed [14], although spotted fever has been monitored for more than a half century [15]. As an emerging tick-borne infectious disease, a survey of farm workers in rural areas of Tianjin has revealed that the average seroprevalence of anaplasmosis among the population is 8.8% [16]. A more recent investigation has reported that the total seroprevalence rates of *E. chaffeensis* and *A. phagocytophilum* among the farm worker population in rural areas of Beijing are 16.4% and 14.1%, respectively [17].

HME and HGA have clinical presentations similar to spotted fever [18], and these 3 tick-borne rickettsial diseases are typically transmitted by tick bites or exposure to ticks. The early signs and symptoms of these illnesses are notoriously nonspecific and mimic benign viral illnesses, making their diagnosis difficult. Misdiagnoses may result in severe multi-organ symptoms or even death in healthy adults and children, despite the availability of low-cost, effective antimicrobial therapy. In this regard, early diagnosis is essential for rapid clinical treatment [19]. However, the conventional PCR technique is time consuming and prone to false-positive results due to contamination. Serological diagnoses based on acute- and recovery-phase sera samples are not generally useful for early diagnosis. Moreover, culture of these organisms is limited to fully equipped, specialized laboratories, such as P3 biosafety laboratories, and such culture systems also require experienced professionals.

Notably, co-infection by *E. chaffeensis* and *A. phagocytophilum* has been frequently reported in both clinical patients and ticks in recent years in China [20]. Therefore, developing a rapid, sensitive and reliable diagnostic test will be valuable for timely diagnosis and treatment. Herein, we describe a duplex real-time PCR assay targeting the *A. phagocytophilum ankA* gene and the *E. chaffeensis* TRP120 gene. A methodological evaluation of the real-time PCR assay demonstrated that the reported real-time PCR results featured high specificity, sensitivity, reproducibility and simplicity. This method could be widely applied to test various samples such as patient and domestic animal blood and tissue samples as well as tick samples, and the specificity, sensitivity and reproducibility of the real-time PCR assay are not influenced by background DNA.

## Materials and Methods

### Design of primers and probes

The *A. phagocytophilum ankA* gene is unique for the members of genus *Anaplasma* and is regarded as one of the most important components in its disease pathogenesis [21,22].

The *E. chaffeensis* TRP120 gene has been demonstrated to be an important immunodominant antigen, and some reports have indicated that the recombinant TRP120 protein is an effective antigen for serological diagnosis [23,24].

For this study, 96 variant sequences (Table S1) of the *A. phagocytophilum ankA* gene and 13 variant sequences (Table S1) of the *E. chaffeensis* tandem repeat region of TRP120, which originated from a variety of host sources and geographic areas of the world, were selected as templates for the design of primers and probes based on the conserved sequences. Primer Premier 5.0 software (PREMIER Biosoft International, 3786 Corina way, Palo Alto, CA) was used for primer design, and ABI Primer Express 3.0 (Applied Biosystems, CA) was used for the TaqMan MGB probe design. The probes were labeled at the 5’ and 3’ ends with 6-carboxyfluorescein (6-FAM) and DPI3, respectively. The detailed sequences and positions of the primers and probes are shown in Table 1. All primers and probes were synthesized by Shanghai GeneCore Biotechnologies Co., Ltd.

**Table 1:** Names and sequences of the duplex PCR primers and probes.

**Name**	**Sequence (5’–3’)**	**Position**	**Length of product (bp)**
**TRP120**			
TRP120-F	AAGATGTTGCGAGTCATGAATCTG	295-318	
TRP120-R	TCCACTTTCTTTTTCTGTTTCTCCTT	379-404	
TRP120-Probe	FAM-ATCAGCCAGCTCAAG-MGB	328-342	110
TRP120-WF^a^	GAACAAGAAAAAACTAACCCTGA	252-274	
TRP120-WR^a^	AAGGTTGAGATACTATTTCATCTTCT	425-450	199
***ankA***			
*ankA*-F	CAGTCGTGAATGTAGAGGGAAAAAC	2561-2585	
*ankA*-R	GGAATCCCCCTTCAGGAACTTG	2671-2692	
*ankA*-Probe	VIC- CGTTCAGCCATCATTGT-MGB	2649-2666	132
*ankA*-WF^b^	TAAAGATGCTTAAAGAGTCTCG	2399-2420	
*ankA*-WR^b^	GCCACTACCCAAGGATGATA	2765-2784	386

^a^ TRP120-WF and TRP120-WR were used to construct reference plasmids for the *E. chaffeensis* 120 kD gene

^b^ *ankA*-WF and *ankA*-WR were used to construct reference plasmids for the *A. phagocytophilum ankA* gene

### Construction of recombinant reference plasmids

To test the sensitivity and reproducibility of the real-time PCR assay, 2 sets of primers, TRP120-WF and TRP120-WD and *ankA*-WF and *ankA*-WR (Table 1), were designed to amplify partial fragments of the *E. chaffeensis* TRP120 gene (199 bp) and the *A. phagocytophilum ankA* gene (386 bp), based on *E. chaffeensis* str. Arkansas (AF474890) and *A. phagocytophilum* str. Webster (GU236811) DNA as a template. The PCR products were purified with a Gel Extraction and PCR Purification Combo Kit (Spin-column, BioTeke Corporation, Beijing), ligated into the pEASY-T1 vector and then transformed into *Trans*1-T1 Phage Resistant Chemically Competent Cells, according to the manufacturer’s instructions (TransGen Biotech, Beijing, China, Cat. No. DP1502). The positive plasmids were then screened by PCR using the specific primers mentioned above, and the recombinant plasmid was purified using a High-purity Plasmid Mini-preparation Kit (Spin-column, BioTeke, Beijing). The recombinant plasmid was further confirmed by commercial sequencing using the general primers for the pEASy-T1 vector (Shanghai Technical Service Industry and Biological Engineering Co., Ltd.). Quantification of the recombinant plasmid was performed using a NanoDrop ND-1000 (NanoDrop Technologies, Wilmington, Delaware). The weight of a single copy of a plasmid and the copy number of the recombinant plasmid per microliter were calculated according to the previously reported formula [25]. A series of 10-fold dilutions of the recombinant plasmids (10^6^, 10^5^, 10^4^, 10^3^, 10^2^ and 10^1^ copies/μl) was used to test the sensitivity and reproducibility of the real-time PCR assay.

### Real-time PCR assay

The duplex PCR assays were performed with an ABI 7500 fast real-time PCR detection system (Applied Biosystems, USA). A total reaction volume of 20 µL contained 10 µL of 2× Premix Ex Taq™ Mix (Takara Bio Premix Ex Taq™), 200 nM of each primer, 200 nM of each probe, 4.4 µL of sterile water and 2 µL of each DNA sample. The cycling conditions were 30 s at 95°C, followed by 40 cycles of 3 s at 95°C and 45 s at 60°C. In addition to sterile distilled water and DNA extracted from the plank pEASy-T1 vector and plank DH82 cells [5,26], which was provided by Dr. Robert Massung at the United Stated CDC, and HL-60 cells [5], which was kindly provided by J. S. Dumler at the Johns Hopkins University School of Medicine; DNA extracted from the blood of healthy human donors, healthy mice and ticks was also used as negative controls.

### Evaluation of the sensitivity, reproducibility and specificity of the real-time PCR assays

To test the sensitivity of the real-time PCR assay, 2 sets of serially diluted reference plasmids (concentrations of 10^6^, 10^5^, 10^4^, 10^3^, 10^2^ and 10^1^ copies/μL) containing the target *A. phagocytophilum ankA* gene and the *E. chaffeensis* TRP120 gene were used as templates to define the lowest detection limits of the real-time PCR assay.

To determine the reproducibility of the real-time PCR assay, concentrations of 10^6^, 10^5^, 10^4^, 10^3^, 10^2^ and 10^1^ copies/μL of each plasmid were individually tested in 3 replicates on separate occasions (three times on one day and once on each of five days). The inter-run variation (CVi%) and intra-run variation (CVo%) were analyzed using Excel software (Table 2).

**Table 2:** Intra-assay (CVi%) and inter-assay (CVo%) variation of the duplex real-time PCR assay.

**Variation**	**Concentration of reference plasmid (copies/µl)**	***E. chaffeensis* 120kD**		***A. phagocytophilumankA***	
		Ct	CV%	Ct	CV%
Intra-assay	1×10^6^	17.2	0.2	18.2	0.9
(CV_O_%)	1×10^5^	20.3	0.9	20.5	0.8
	1×10^4^	23.9	1.3	24.3	0.1
	1×10^3^	27.6	0.7	28.1	1
	1×10^2^	31.6	0.6	31.7	1.1
Inter-assay	1×10^6^	17.5	3.5	18.9	2.6
(CV_I_%)	1×10^5^	20.4	2.9	21.9	3.1
	1×10^4^	24.2	3.2	25.2	2.7
	1×10^3^	27.7	3.3	28.3	3.7
	1×10^2^	31.8	2	32.6	2

Twenty-seven members of the order Rickettsiales, 3 related strains and 13 common clinical pathogens were used to determine the specificity of the real-time PCR assay. The strains included the following: *Rickettsia prowazekii;* *Rickettsia* *typhi* *;* *Orientia* *tsutsugamushi* types Karp, Kato, and Gilliam; *Rickettsia* *sibirica* *; Rickettsia conorii;* *Rickettsia* *marmionii* *;* *Rickettsia* *akari* *; Rickettsia rickettsii;* *Rickettsia* *africa* *;* *Rickettsia* *parkeri* *;* *Rickettsia* *japonica* *;* *Rickettsia* *slovaca* *;* *Rickettsia* *aeschlimannii* *; Rickettsia montanensis;* *Rickettsia* *helvetica* *;* *Rickettsia* *felis* *;* *Rickettsia* *australis* *;* *Rickettsia* *canadensis* *;* *Rickettsia* *bellii* *; Rickettsia heilongjiangensis*; 3 related species, *Bartonella henselae, Bartonella quintana* and *Coxiella burnetii* (kindly provided by the Dr Didier Raoult at the WHO Collaborative Center for Rickettsial Reference and Research, Marseille, France); *Anaplasma phagocytophilum* strains Webster, MRK, Slovenia and MD, which were gifts of Dr. J. S. Dumler at the Johns Hopkins University School of Medicine; *Ehrlichia chaffeensis*, which was provided by Dr. Robert Massung from the U.S. CDC; 13 common clinical pathogenic agents, including *Borrelia burgdorferi, Escherichia coli, Vibrio cholerae, Bacillus anthracis,* *Haemophilus* *inﬂuenzae* *,* *Listeria* spp., *Legionella* spp., *Yersinia pestis, Shigella dysenteriae,* *Neisseria* *meningitidis* *,* *Leptospira* spp., *Mycobacterium tuberculosis* and *Klebsiella pneumoniae* (provided by the relevant laboratories, National Institute for Communicable Disease Control and Prevention, China CDC). DNAs extracted from the blood of healthy humans and animals, including goats, cats, dogs, cattle, horses, mice and ticks (previously prepared and conserved in our laboratory), was also tested as negative controls. A total of 3 µL of each DNA sample was assayed by the real-time PCR assay.

### Background DNA interference

To detect interference by eukaryotic background DNA in the various samples, serially diluted eukaryotic DNA, including DNA from the blood of humans, goats, sheep, cats, dogs, cattle, horses, mice and ticks (concentrations of 50 ng, 25 ng, 12.5 ng, 6.3 ng, 3.1 ng and 1.5 ng per reaction), were added to produce individual concentrations of 10^6^, 10^5^, 10^4^, 10^3^, 10^2^ and 10^1^ copies/μl of the reference plasmids, respectively. The DNA dilutions were tested as a template by real-time PCR to observe whether the sensitivity and reproducibility of the real-time PCR assay had changed.

### Clinical sample collection and detection

#### Ethic statements

The ethics committee of the China CDC reviewed and approved the study protocol (No. 201103). A written consent form was obtained before collecting blood from the patients. All experimental procedures with wild animals were conducted to conform to the National Institutes of Health Guide for Care and Use of Laboratory Animals (Publication No 85-23, revised 1985). The Animal Ethics Committee of the Chinese Center for Disease Control and Prevention approved the document on experimental procedures (201104). Sampling blood of domestic animals was conducted when the owners of the animals gave permission for their animals.

#### Collection and detection of HGA and HME cases

Blood samples from 22 HGA patients and 8 HME patients were collected as part of a collaborative project between the Department of Anaplasma, National Institute for Communicable Disease Control and Prevention, China CDC, and five clinical hospitals, including Peking University First Hospital, Laizhou People’s Hospital, Shandong Province, Yantai Infectious Diseases Hospital, Shandong Province, the Dept. of Infectious Disease 302 Military Hospital, Beijing, China, and the Tianjin CDC in 2009-2012 [27]. Patient blood samples were centrifuged to isolate serum for the serological assay (micro-indirect immunofluorescence assay (IFA)) [28], and the remaining blood clot was used to extract DNA (QIAGEN, Germany, Cat. No. 69506) for molecular detection using real-time PCR and nested PCR [29]. Patient blood samples were stored temporarily at -20^°^C at local clinical laboratories, and the samples were transported to the Department of Anaplasma, National Institute for Communicable Disease Control and Prevention, China CDC, within 24 hours. In addition, 17 healthy human sera and blood DNA, which were collected from research members of the institute during health examinations, were used in the IFA, real-time PCR and nested PCR as negative controls.

The HGA and HME cases were diagnosed serologically using IFA and nested PCR targeting the 16S rRNA gene of *E. chaffeensis* and *A. phagocytophilum* and pathogenic bacteria isolation (Table 3). The 22 HGA cases were confirmed by a fourfold increase in the specific IgG antibody titer. Of these cases, 3 patients’ blood DNA samples were found to be positive by the nested PCR assay, and 4 patients were found to be positive by bacterial culture. For the 8 HME cases, all patients were diagnosed by a fourfold elevation of the IgG titer. Of these cases, only 1 was found to be positive by the nested PCR assay (Table 3).

**Table 3:** Laboratory diagnosis data for 22 HGA cases and 8 HME cases.

**Region**	**Patient or healthy personr healthy**	**No.**	**4-fold increase in IgG titer (%)**		**Nested PCR%(No. positive / No. tested)**		**Duplex real-time PCR%(No. positive / No. tested)**		
			***A. phagocytophilum***	***E. chaffeensis***	***A. phagocytophilum***	***E. chaffeensis***	***A. phagocytophilum***	***E. chaffeensis***	***Co-infection***
Beijing	HGA	8	100		12.5 (1/8)	0	12.5 (1/8)	0	0
	HME	2		100	0	50 (1/2)	0	50 (1/2)	0
Tianjin	HGA	2	100		0	0	0	0	0
	HME	2		100	0	0	0	50 (1/2)	0
Shandong	HGA	12	100		16.7 (2/12)		33.3 (4/12)	0	0
	HME	4		100		0	0	0	0
Total		30	-		13.6 (3/22)	12.5 (1/8)	22.7 (5/22)	25.0 (2/8)	0
Beijing (China CDC)	Healthy personsrson	17	N^a^	N^a^	0	0	0	0	0

N^a^: The specific IgG antibody titer for each person was minor, 1:80

### Tick and wild mouse collection

Tick and wild mouse samples are summarized in Table 4. *Citellus* *undulatus* Pallas spleen samples were collected in Yili areas, Xinjiang Province in 2009, which is a non-national protection area of land and wildlife and the field investigation and sampling was approved by the YiLi Prefecture Center for Disease Control and Prevention, Xinjiang Province. All samples were stored temporarily at -20^°^C at local CDC laboratories. A total of 594 *D* *. silvarum* ticks were collected in 2011 in Ningwu County, which is non-national forest and wild animal reserve, Shanxi Province, and the Shanxi provincial CDC approved the activity. A total of 570 *H. longicornis* (114 questing and 456 from domestic animals, including sheep, goats, cattle and dogs) were collected in Laizhou Bay, Shandong Province in 2010; the questing ticks were obtained from a farm family plot and the domestic animals were also owned by farmers. Sampling of questing ticks and from animals was approved by the owners of the land and domestic animals. Ticks were stored temporarily in a clean container at -4^°^C at local clinical laboratories. All samples were transported to the Department of Anaplasma, National Institute for Communicable Disease Control and Prevention, China CDC, within 24 hours. The ticks were disinfected by immersion in a 75% ethanol solution for 30 minutes and then rinsed with sterile water 3 times for 10 minutes each. The ticks were divided into blood-engorged and non-blood-engorged groups for grinding, using a Retsch MM 400 mixer mill. Tick sample pools (61 *D* *. silvarum* pools from Ningwu County, Shanxi Province, and 64 *H. longicornis* pools from Laizhou Bay, Shandong Province) were then subjected to DNA extraction using the same reagents described in the procedure for the extraction of DNA from patient blood. In total, 3 µL of genomic DNA was used as the template for the PCR assay. Sterile water and blood samples from healthy humans, dogs, horses, cattle, rabbits, mice, sheep, goats and ticks were subjected to the same DNA extraction process, and all of these extracted DNA samples were used as negative controls.

**Table 4:** Detection in ticks and mice by nested PCR and duplex real-time PCR.

**Region**	**Specimen**	**No.**	**IFA %(Positive No. /tested No.)**			**Nested PCR %(Positive No. /tested No.)**		**Duplex real-time PCR %(Positive No. /tested No.)**		
			***A. phagocytophilum***	***E. chaffeensis***	**Co-infection**	***A. phagocytophilum***	***E. chaffeensis***	***A. phagocytophilum***	***E. chaffeensis***	**Co-infection**
Yili, Xinjiang	*Citellus* *undulatus* * Pallas*	32	6.3 (2/32)	6.3 (2/32)	3.1 (1/32)	0	0	3.1 (1/32)	6.3 (2/32)	3.1 (1/32)
Ningwu County, Shanxi	*D* *. silvarum*	594/61 pools	_	_	_	57.3 (35/61)	9.8 (6/61)	8.2 (5/61)	14.8 (9/61)	3.3 (2/61)
Laizhou, Shandong	*H. longicornis*	570/64 pools	_	_	_	45.3 (29/64)	3.1 (2/64)	1.6 (1/64)	6.3 (4/64)	1.6 (1/64)

## Results

### Sensitivity, reproducibility and specificity of the duplex real-time PCR

The lowest detection limit of the developed duplex real-time PCR for *A. phagocytophilum* and *E. chaffeensis* was 100 copies per reaction for each bacterial species. The correlation coefﬁcient (R) between the Ct values and the reference plasmid concentration was -0.998, demonstrating high quantification accuracy.

The mean intra-assay variation (CVi%) and inter-assay variation (CVo%) were 2.8% (2.0-3.7%) and 0.8% (0.1-1.1%), respectively, for the detection of the *A. phagocytophilum ankA* gene, and they were 3.0% (2.1%-3.52%) and 0.7% (0.2-1.3%), respectively, for the detection of the *E. chaffeensis* TRP120 gene (Table 2).

With the exception of *E. chaffeensis* and *A. phagocytophilum* types Webster, MRK, Slovenia and MD, the other 27 members of order Rickettsiales were negative by the duplex real-time PCR assay. In addition, the 13 common clinical pathogens mentioned in the materials and methods as well as healthy human and animal blood DNA (including goat, sheep, cat, dog, cattle, horse, and mouse and tick genomic DNA) were also negative.

### Background DNA interference

The interfering background DNA test indicated that the sensitivity (lowest detection limits) of the real-time PCR was not affected by the eukaryotic DNA of healthy humans, goats, sheep, cats, dogs, cattle, horses, mice or by tick DNA.

### Examination of samples

Using the developed real-time PCR assay, we evaluated 22 blood DNA samples from HGA patients (acute phase of illness) and 8 blood DNA samples from HME patients; all cases were confirmed in the serological assay (IFA, 4-fold increase in the IgG titer in acute- and convalescent-phase serum samples). The results showed that 22.7% (5/22) of HGA patients and 25.0% (2/8) of HME patients were positive. Using the nested PCR assay, 13.6% (3/22) and 12.5% (1/8) of the confirmed cases were positive for *A. phagocytophilum* and *E. chaffeensis*, respectively (Table 3). All of the healthy human sera and blood DNA were negative by IFA and the two PCR methods, respectively. Similarly, *Citellus* *undulatus* Pallas infection by *A. phagocytophilum* and *E. chaffeensis* was confirmed for the first time in Xinjiang Province by the real-time PCR assay. The PCR positive rate was 3.1% for *A. phagocytophilum* and 6.3% for *E. chaffeensis*, and the co-infection rate was 3.1% for *A. phagocytophilum* and *E. chaffeensis*, which was further confirmed by serological IFA. The IFA positive rate was 6.3% for *A. phagocytophilum* and 6.3% for *E. chaffeensis*, and the co-infection rate was 3.1% (Table 4). The positive rates of the 61 *D* *. silvarum* sample pools, which were collected from Ningwu County, Shanxi Province, were 8.2% for *A. phagocytophilum* and 14.8% for *E. chaffeensis*, and the rate of co-infection by both pathogens was 3.3%. Among the 64 *H. longicornis* ticks collected from the Laizhou Bay area, Shandong Province, 1.6% was positive for *A. phagocytophilum*, and 6.3% were positive for *E. chaffeensis*; and the co-infection rate was 1.6% (Table 4).

## Discussion

*E. chaffeensis* TRP120 proteins are strongly recognized by the host immune response, and recombinant TRP120 protein is considered to be a valuable diagnostic antigen. More recent data have indicated that the TRP120 protein is involved in ehrlichial molecular pathogenesis by modulating host gene expression to facilitate its intracellular survival and proliferation [30,31]. Similarly, *A. phagocytophilum ankA* is an important protein in disease pathogenesis, and it can alter transcription by binding specific nuclear proteins, resulting in a series of neutrophil functional alterations [3,21,22].

In our study, a rapid, sensitive and speciﬁc duplex real-time PCR assay was developed for the simultaneous diagnosis HGA and HME based on the *A. phagocytophilum ankA* gene and the *E. chaffeensis* TRP120 gene. The detection limit was 100 copies of the target DNA per reaction, which is similar to some previous reports [32,33]. High speciﬁcity (100%) was observed when we performed the duplex real-time PCR assay on 27 members of the order Rickettsiales, 3 related bacteria and 13 common clinically pathogenic bacteria. Perfect reproducibility was observed when evaluating intra- and inter-assay variation in parallel (Table 2). The presence of background eukaryotic DNA, including healthy human, goat, sheep, cat, dog, cattle, horse, mouse and tick DNA, did not affect the sensitivity or reproducibility of the real-time PCR assay. Using DNA extracted from the blood of 22 confirmed HGA patients and 8 confirmed HME patients during the acute-phase of illness, we evaluated the sensitivity of the duplex real-time assay, which was higher than that of the nested PCR assay [29]. The positive rates of the duplex real-time PCR were 22.7% and 25.0% for HGA and HME, respectively, while the nested PCR positive rates were 13.6% and 12.5%, respectively. The probable reason for the disagreement between the conventional PCR and the real time PCR was probably due to the interfering action that occurs with more background DNA used in the conventional PCR (10 µL template DNA for the nested PCR vs. 3 µL template DNA for the real time PCR).

*Citellus* *undulatus* Pallas (the dominant local wild mouse species) infection by *A. phagocytophilum* and *E. chaffeensis* in Xinjiang Province was first confirmed by serological and molecular detection. Additionally, we are the first to identify the co-infection of the two tick-borne zoonotic rickettsiae in *D* *. silvarum* and *H. longicornis*, which are the dominant tick species in the forested areas of China. The co-infection rate was 3.3% for *D* *. silvarum* and 1.6% for *H. Longicornis*, respectively. Recently, 2.5% and 0.7% of *D* *. silvarum* collected from China–Russia border [34] and the forested areas of Jinlin Province in China [35], respectively, have been reported to be infected by *A. phagocytophilum*. *H. longicornis* is distributed nationwide and is the dominant parasitic tick for most domestic animals in China. In 2009-2010, 2 human isolates and 1 tick isolate of *A. phagocytophilum* were obtained from patients and *H. longicornis* ticks collected from the local patients’ domestic animals in Laizhou Bay, Shandong Province, respectively [27]. A more recent study demonstrated that 3.3% of *H. longicornis* ticks collected from the China–Russia border were infected by *A. phagocytophilum* [34]. In China, infection of *H. longicornis* and *D* *. silvarum* by *E. chaffeensis* has rarely been reported, with the exception of this study.

The evidence from this study and previous studies indicates that these two emerging tick-borne zoonotic rickettsiae are commonly distributed in ticks in China [13,16,17,36]. The diagnosis of these infectious diseases can be challenging, especially during the acute phase. PCR assays for *E. chaffeensis* and *A. phagocytophilum* have been developed in our laboratory before. These assays include conventional PCR and single color real time PCR. However, these PCR assays involve time-consuming two round reactions of nested PCR and post-amplification detection or 2 separate individual single color real time PCR performance on *E. chaffeensis* and *A. phagocytophilum* respectively. The duplex real-time PCR assay established in this study can be capable of the simultaneous detection and differentiation of *A. phagocytophilum* and *E. chaffeensis* and this will greatly improve the detection of these two emerging tick-borne pathogens in various samples, particularly the timely screening of clinical samples from patients with an unknown febrile illness and field samples in endemic areas.

## Supporting Information

Table S1 **Reference sequences used to design the primers and probes for duplex real-time PCR.** A total of 96 variant sequences of the *A. phagocytophilum ankA* gene from a variety of host sources and geographic areas of the world, and 13 variant sequences (12 from human and 1 from white-tailed deer) of the *E. chaffeensis* tandem repeat region of TRP120 were selected for the design of primers and probes based on the conserved sequences.
(DOC)
