# Supplementary material for: Detection of A. phagocytophilum and E. chaffeensis in Patient and Mouse Blood and Ticks by a Duplex Real-Time PCR Assay
Source: PLoS One. 2013 Sep 4;8(9):e74796. doi: 10.1371/journal.pone.0074796 (PMC3762869; doi:10.1371/journal.pone.0074796)
Supplement: Table S1 — Reference sequences used to design the primers and probes for duplex real-time PCR. A total of 96 variant sequences of the A. phagocytophilum ankA gene from a variety of host sources and geographic areas of the world, and 13 variant sequences (12 from human and 1 from white-tailed deer) of the E. chaffeensis tandem repeat region of TRP120 were selected for the design of primers and probes based on the conserved sequences. (DOC) [file pone.0074796.s001.doc]

**Table S1.** Reference sequences used to design the primers and probes for duplex real-time PCR

| **Gene** | **Origen** | **GenBank accession number (Region)** |
| --- | --- | --- |
| ***E. chaffeensis* 120 kD** | Human(12) | AF474890(USA: Florida, Osciola), AF474891(USA: Georgia, Lithonia), AF474892(USA: Tennessee, Chattanooga), AF474893(USA: Tennessee, West Paces), AF474894(USA: Nebraska, Heartland), AF474895(USA: Florida, Liberty), AF474896(USA: Florida, Wakulla), AF474897(USA: Florida, Jax), AF474898(USA: Arkansas), AF474899(USA: Georgia, St. Vincent), U74670(USA: TX, Galveston ) CP000236(USA: Rockville) |
|  | White-Tailed Deer(1) | AY307354(USA: Chatham Co., GA) |
| ***A. phagocytophilum ankA*** | Human (12) | GU236800(Slovenia) GU236801(Slovenia) GU236802(Slovenia) GU236803(Slovenia) GU236804(Slovenia) GU236805(Slovenia) GU236806(USA) GU236807(USA) GU236808(USA) GU236809(USA) GU236810(USA) GU236811(USA) |
|  | Dogs (43) | GU236812(Slovenia) GU236813(Slovenia) GU236814(Slovenia) GU236815(Slovenia) GU236816(Slovenia) GU236817(Austria) GU236818(Denmark) GU236819(Germany) GU236820(Germany) GU236821(Sweden) GU236822(Sweden) GU236823(Germany) GU236824(Germany) GU236825(Germany) GU236826(Austria) GU236827(Germany) GU236828(Germany) GU236829(Germany) GU236830(Germany) GU236831(Germany) GU236832(Germany) GU236833(Germany) GU236834(Germany) GU236835(Germany) GU236836(Germany) GU236837(Germany) GU236838(Germany) GU236839(Germany) GU236840(Germany) GU236841(Germany) GU236842(Germany) GU236843(Germany) GU236844(Germany) GU236845(Germany) GU236846(Switzerland) GU236847(Germany) GU236848(Switzerland) GU236849(Germany) GU236850(Switzerland) GU236851(Germany) GU236852(Switzerland) GU236853(Germany) GU236854(Germany) |
|  | Sheep (12) | GU236755(Norway) GU236757(Norway) GU236758(Norway) GU236759(Norway) GU236767(Norway) GU236768(Norway) GU236771(Norway) GU236791(Germany) GU236793(Germany) GU236795(Germany) GU236796(Germany) GU236799(Norway) |
|  | Cows (3) | GU236729(Spain) GU236730(Spain) GU236731(Norway) |
|  | Cats (2) | GU236864(Austria) FJ515309d(Switzerland) |
|  | European bison ( 9) | GU236733(Poland) GU236734(Poland) GU236735(Poland) GU236736(Poland) GU236738(Poland) GU236739(Poland) GU236742(Poland) GU236744 (Poland) GU236745(Poland) |
|  | Red deer (5) | GU236718(Slovenia) GU236719(Slovenia) GU236722(Slovenia) GU236725(Poland) GU236727(Germany) |
